# Dietary Interventions to Promote Healthy Eating among Office Workers: A Literature Review

**DOI:** 10.3390/nu12123754

**Published:** 2020-12-07

**Authors:** Alkyoni Glympi, Amalia Chasioti, Katarina Bälter

**Affiliations:** 1 School of Health, Care and Social Welfare, Division of Public Health Sciences, Mälardalen University, 722 20 Västerås, Sweden; amaliaxas@hotmail.com (A.C.); katarina.balter@mdh.se (K.B.); 2 Department of Medical Epidemiology and Biostatistics, Karolinska Institutet, 171 77 Stockholm, Sweden

**Keywords:** dietary intervention, office workers, healthy diet, dietary behavior

## Abstract

Our aim is to review published studies on dietary interventions to promote healthy eating habits among office workers. The databases PubMed, EBSCO (MEDLINE, Academic Search Elite, CINAHL Plus, PsycARTICLES, PsycINFO), Cochrane Library, SCOPUS, and Google Scholar were searched between February and April 2019. Initially, 6647 articles were identified, and the final number of articles that met the inclusion criteria was 25. We identified four different types of interventions that included educational and/or environmental components, where environmental components provided healthy food in a work-related context. The interventions at the offices included web-based material, availability of food, provision of information in various ways, and a combination of environmental, educational and theory-based psychological approaches (i.e., multicomponent). The most commonly used designs were web-based and information interventions, respectively, which are the least expensive ways to intervene. The interventions assessed a range of outcomes, but this literature review focused on three, i.e., dietary intake, dietary behavior and health-related outcomes. Although the studies were heterogenous in terms of outcomes, design, number of participants, gender distribution and duration, all studies reported at least one positive effect. Thus, workplace dietary interventions are an unutilized area to positively influence dietary intake and health outcomes among office workers. However, the intervention needs to be tailored to the workplace.

## 1. Introduction

Non-communicable diseases (NCDs) such as obesity, cardiovascular diseases (hypertension, stroke, and atherosclerosis), various types of cancer (breast, colon, and prostate), and type II diabetes are the leading cause of death globally, and one of the major health challenges of the 21st century [1]. Every day, 3.45 billion people go to work [2] and NCDs contribute 70%, injuries 22% and infectious diseases 8% to the total disease burden from occupational risks [3].

More than half of the employees in Sweden work in offices, [4] and the situation is the same in many other developed countries (e.g., Germany) [5]. Working in an office setting is characterized by sedentary work, and the main components associated with the development of NCDs are physical inactivity and poor food habits [6]. An office worker is defined as “an employee who works in an office, especially one engaged in clerical or administrative work” [7]. Another term for this type of work is white-collar workers and they are defined as “people who work in offices, doing work that needs mental rather than physical effort” [8]. Coincidently, the retirement age has increased, and people need to work for more years and at older ages, including an age where age-related NCDs are getting more common. The general retirement age in the EU Member States is 65 years. Germany, Spain and France are about to raise their retirement age from 65 to 67 years, while, in Ireland and Britain, the aim is 68 years. Furthermore, due to the increase in life expectancy, the retirement age in many countries such as Cyprus, Estonia, Denmark, Italy, Greece, Portugal, the Netherlands and Slovakia will increase as well between 2020 and 2030 [9]. Thus, it is crucial that precautionary actions are taken to prevent the development of NCDs and to help manage early stages of NCDs by offering healthy lifestyles at work.

The workplace is and can be one of the most important settings affecting eating habits and the physical, mental and social well-being of office workers. A substantial proportion of the adult population spends a significant amount of time at work each day and a large number of individuals can be targeted simultaneously in a work office-based intervention. Moreover, these types of interventions may be advantageous because they are convenient and easily accessible to workers and allow employees to interact and support each other in their efforts to undertake behavior changes [10]. Full-time employees spend up to 60% of their waking hours at work [11] and typically return repeatedly to the same location, providing a significant opportunity to deliver health interventions to a target population. Thus, office-based wellness programs may be an ideal large-scale strategy to promote healthy behaviors to combat this rise in NCDs. Moreover, the health care system and employers may benefit from reduced incidence of NCDs among office workers since a considerable amount of tax money is spent on treating chronic diseases and companies are suffering from productivity losses [12].

To the best of our knowledge, this is the first literature review comprising published studies worldwide focusing on either dietary or multicomponent lifestyle interventions among office workers. Here, we present the effects on diet, dietary behavior and health-related outcomes for various types of interventions, and the results may be implemented in office contexts.

## 2. Methods 

This literature review aims to investigate and describe the published up-to-date data on the effectiveness of dietary interventions for promoting healthy eating habits and healthy lifestyles among office workers.

### 2.1. Inclusion Criteria

The following criteria were applied in the literature search of published research articles: the articles had to (1) be published between 1999 and 2019; (2) be published in the English language; (3) be interventions in an office setting; (4) explicitly describe dietary intervention components or the impact of a multicomponent intervention on diet-related outcomes (changes in dietary intake); (5) describe the effect of dietary intervention components on dietary behavior-related outcomes (i.e., knowledge, attitude and skills related to dietary intake) and health-related outcomes (i.e., weight, BMI, waist circumference, blood pressure, blood lipids, fasting blood glucose); (6) include healthy office workers as well as office workers with overweight, metabolic syndrome and potentially other health problems; and (7), for studies with environmental intervention, the intervention must have been conducted within a workplace, or frequented by office employees for eating.

No limitations were set as to the subject characteristics (e.g., gender), worksite size (e.g., number of employees), follow-up measurements (e.g., short-term, long-term), control group (e.g., health risks appraisal (HRA), waiting list, and no intervention), type of intervention (e.g., environmental, educational, and multicomponent) or study design. We included intervention studies with different designs, including randomized controlled trials and before–after designs at a single site, in order to capture as many relevant studies as possible to study the effectiveness of dietary interventions in an office setting. Studies without an intervention component (e.g., observational) and non-dietary interventions (e.g., exercise) were excluded.

### 2.2. Data Sources/Literature Search 

Articles were identified by searching five electronic databases and reviewing reference lists of articles, relevant review articles, recommended articles, and meta-analysis. Recommended articles were retrieved from the option “similar articles” in PubMed. This search was applied to PubMed, EBSCO (MEDLINE, Academic Search Elite, CINAHL Plus, PsycARTICLES, PsycINFO) Cochrane Library, SCOPUS, and Google Scholar. A time restriction was applied for articles published between 1 January 1999 and 15 April 2019, and the final search was done on 16 April 2019. 

The following keywords were used in various combinations to search all databases: “office worker*” OR “office employees*” OR “white collar*” OR “sedentary occupation” OR “sedentary job” AND “healthy eating” OR “healthy diet” OR “healthy nutrition” OR “healthy eating behavior” AND intervention* OR program* AND “worksite health promotion” OR “workplace health promotion”. 

Due to the small number of identified articles when using the keywords, the majority of articles were found via the feature recommended articles in PubMed and by screening the reference lists of all previously identified articles using a snowballing approach.

### 2.3. Search Outcome

The search in five databases, the recommended articles in PubMed and the reference lists provided a total of 6647 articles. From the initial review, 5598 articles were discarded after screening titles. Eight hundred fifty-one articles of the remaining 1049 were removed after a review of abstracts since they did not meet the inclusion criteria, i.e., it was not clear from the abstract if they included office workers and/or a dietary intervention. A total of 198 full-text articles were assessed for eligibility and 95 remained after adjusting for duplicates.

**Figure 1:**
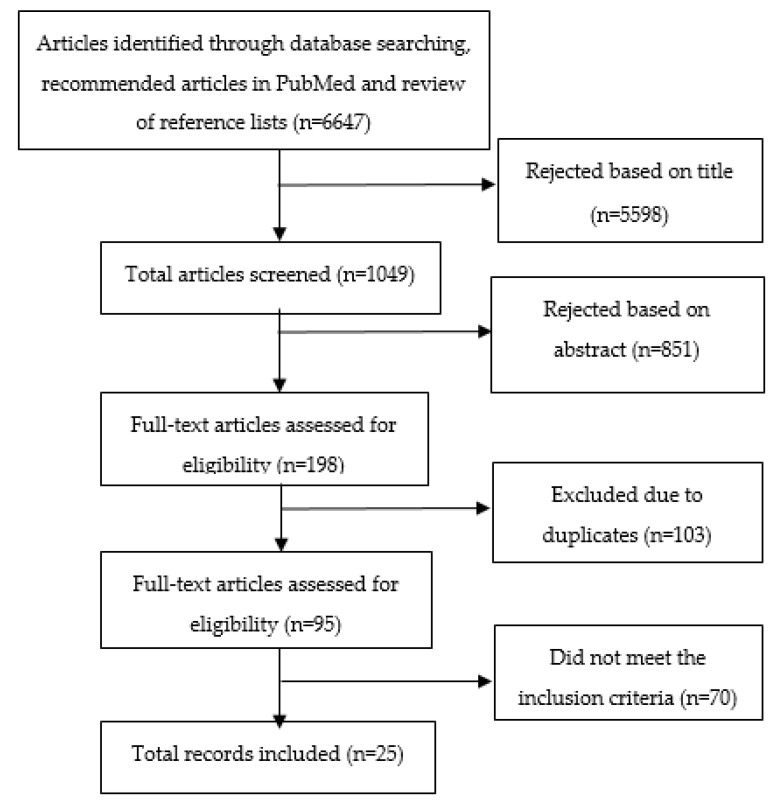
Flow chart of the article selection process.

The full text of the remaining 95 articles was read in more detail and 70 studies did not meet the inclusion criteria. These articles were rejected for several reasons, for example, some studies included a combination of white- and blue-collar workers, some studies were not intervention studies and some did not have diet as the main focus. A total of 25 articles from 17 unique trials were identified for inclusion in the present review; see the selection process in Figure 1. Four of these final articles were identified in the database screening with keywords and 21 articles were identified by checking the reference lists and relevant articles in PubMed. No unpublished studies were obtained.

### 2.4. Study Selection

Two reviewers (AC and AG) independently applied the inclusion criteria to select potentially relevant articles from the titles, abstracts and keywords of the references retrieved from the literature search and the reference lists of articles, relevant review articles, recommended articles, and meta-analysis. Disagreements between reviewers were resolved by consensus. The inclusion criteria were pilot tested by both reviewers on one article that was not included in this review, to resolve the initial disagreement.

### 2.5. Data Extraction/Data Collection Process

Data were independently extracted by the two reviewers using a data extraction form developed by us. Each study was summarized regarding the author and the year, the study design, follow-up duration, sample size, intervention type, intervention, incentive, outcome measures, and results, and listed in Table 1, Table 2, Table 3 and Table 4. These tables focus on positive findings from the interventions, since the aim of the review paper is to highlight the potential of office based dietary interventions and not to compare studies. The data extraction form was pilot-tested on two articles. Disagreements were resolved by discussion between the two review authors.

## 3. Results

### 3.1. Characteristics of Included Studies

Among the 25 identified studies, 14 were intervention studies aimed exclusively at improving diet or dietary behavior [6,15,16,18,20,21,22,23,24,25,31,32,34], while the rest focused on a combination of dietary and other health-related behaviors such as physical activity, quitting smoking, reducing stress, etc.

Outcome measures were self-reported dietary intake (e.g., fruit and vegetable and fat consumption), attitudes toward a healthy diet (e.g., nutrition knowledge, psychosocial determinants of behavior), physiological outcomes (e.g., weight, body fat% and body mass index), physical activity and clinical markers such as cholesterol, blood sugar and blood pressure as well as sales data in canteens. In this review, we considered health-related outcomes as a broader category which incorporates physiological outcomes and clinical markers, as both of them are associated with the onset of NCDs [1].

The interventions included a range of different study designs. Thirteen were randomized controlled trials [12,13,16,17,19,23,25,26,27,28,31,33,34] and the rest were interventions with various designs including controlled trials (longitudinal, non-randomized), non-randomized observational studies, quasi-experimental and pretest-posttest evaluations. Regardless of the design, all studies included an intervention and a control group, except for five [6,14,15,20,29] which examined the differences within the intervention group over time without having a control group.

Less than half of the studies (10 out of 25) applied a behavior change theory to facilitate health and food behavior change. The social cognitive theory (SCT) was used in three interventions [12,16,17], the transtheoretical model of behavior change (TMBC) in another three [14,17,18] and the social ecological model (SEM) in two [25,34]. Finally, one study applied the health belief model [10], one the ‘attitude-social influence- (self-) efficacy model’ (ASE model) [24] and one the theory of planned behavior (TPB) [23].

The number of participants ranged from 22 to 1294 and the length of the intervention varied from four weeks to 19.5 months. Four studies included male participants only [6,22,27,33], two studies included only female participants [16,18] and the remaining 19 included both male and female participants. Among them, 13 had mainly female participants [10,13,14,17,19,20,21,23,25,26,28,29,34], while six had mainly male participants [12,15,24,30,31,32]. Twelve studies were conducted in the USA, nine in Europe (three in Denmark, two in the UK and four in the Netherlands), one in Korea, one in Australia and two in Japan. 

Based on the description of characteristics, 10 studies [6,12,14,17,22,23,24,25,30,33] specifically mention the type of work as “office work” while the other 15 did not clearly state the term “office workers”. In particular, seven studies include company employees [15,17,19,20,27,28,32], five university employees [10,16,18,26,29], three white-collar employees in health care organizations [13,14,34] and two incorporate blue and white collar workers with a higher percentage of white collars [21,31]. For a summary of study characteristics, see Table 1, Table 2, Table 3 and Table 4.

The literature search identified four different types of interventions that consisted of educational and environmental components, where environmental components were mainly providing healthy food in a work-related context. The interventions at the offices included web-based material, availability of food, provision of information in various ways, and a combination of environmental, educational and theory-based psychological approaches (i.e., multicomponent). These interventions assessed a range of outcomes, but this literature review focused only on three, i.e., dietary intake, dietary behavior and health-related outcomes (weight, Body Fat%, BMI, clinical markers such as cholesterol, blood sugar and blood pressure).

### 3.2. Types of Interventions

#### 3.2.1. Web-Based Interventions

Eight of the 25 studies implemented a web-based intervention to promote healthy dietary practices [12,13,14,15,16,17,18,19]. The main components of this type of intervention were the provision of information and guidance on the major health promotion topics (educational sessions and material, webinars, advice, feedback, goal setting, articles, messages, books, newsletters, recipes, etc.). In addition to the basic intervention components, some participants received nutrition consultations via telephone [15] and an eating plan [15,16]. In five studies, self-reported data on dietary intake and lifestyle were collected [12,13,14,16,17]. In two studies, self-reported dietary intake data were collected along with measurements of anthropometric and clinical data conducted by qualified staff [18,19] and, in one study, qualified staff measured indirectly dietary intake via body composition and skin carotenoid level (biomarker for fruit and vegetable intake) [15].

Outcomes under review were diet, dietary behavior change, physical activity, body composition (body weight, percent of body fat, visceral fat level (cm^2^)), clinical biomarkers (blood pressure, fasting plasma lipids, etc.), aging beliefs, tobacco use, stress, and mental health.

Of the seven studies which collected data on diet outcomes, four studies found positive effects [13,14,16,18], of which one was based on pre- and post-assessment within the same group [30]. Improvements included increased intake of fruits and vegetables [13,18,30], reduced intake of saturated, trans fats and added sugars [13], increased intake of dairy products [16], and increased consumption of nuts, seeds, legumes and the monounsaturated fatty acids (MUFA): saturated fatty acids ratio (SFA)ratio [18].

Improved dietary behavior was reported in two studies [12,19] expressed as improvements in diet behavioral change self-efficacy (how confident they are that they can change their dietary practice), planning healthy eating (if they have a good plan for “maintaining a nutritious diet” and for “minimizing the amount of fats and sugars in my diet.”) [12], dietary attitudes and dietary self-efficacy [19].

Positive changes in health-related outcomes (changes in body composition or clinical biomarkers) were observed in four studies [15,16,18,19].

#### 3.2.2. Intervention Where Healthy Food Is Provided

Three of the 25 studies provide healthy food as a means to improve dietary habits and the health status of the workers [20,21,22]. Two strategies were used to achieve this goal; increased availability of healthy foods and provision of a ready-made meal with a predefined nutritional composition. The main food items made available at workplaces were free fruits placed in common areas [21], a balanced Japanese-style healthy lunch with sufficient amount of vegetables [22], and a canteen take-away meal for employees as well as for their families [20]. The assessments that the participants had to undertake were mainly anthropometric measurements [20,21,22], blood parameters [22] and 24h dietary recall [20,21,22]. Two studies included self-reports of food intake [20,21] and one self-report of food intake and measurements conducted by qualified staff [22].

Outcomes included food intake (fruits, vegetables, fiber, protein, fat, added sugar), nutrient intake (energy density, carbohydrate) and clinical biomarkers (total cholesterol, Low-Density Lipoprotein (LDL), blood pressure, etc.).

Improvements in dietary intake were reported in all three studies and comprised of increased intake of fruit, vegetables [20,21,22], fiber [21,22] and proportion of energy coming from protein [20]. Moreover, decreases occurred in energy density [20], added sugar [21], total energy, and carbohydrate intake [22].

Only the study in which Japanese-style healthy lunches were provided examined health-related outcomes and found positive effects on blood parameters. In the group that ate the Japanese-style healthy lunch menu less than 50 times out of the total 61 times, plasma active ghrelin and desacylghrelin levels significantly increased and diastolic blood pressure significantly decreased. In the group that ate this lunch menu more than 51 times out of the total 61 times total cholesterol, LDL, levels significantly decreased and body fat percentage, systolic blood pressure and diastolic blood pressure significantly decreased [22].

#### 3.2.3. Intervention Providing Information

Nine of the 25 included studies were limited to providing information to promote healthy dietary practices. Information was given by means of nutrition education sessions and counseling [6,10,25,26,27,28,29], information sheets, brochures and leaflets [24,30] and placement of nutrition logo on cafeteria menu items [23]. Two studies included self-reports (dietary intake, health beliefs, and nutrition knowledge) [10,24], four contained data from self-reports (lifestyle parameters, health behavior, mental health, mindfulness) and measurements (anthropometric, clinical and exercise) conducted by qualified staff [6,27,28,29]. Another study included sales data from 25 worksite cafeterias and self-reports the participants’ BMI, behavioral determinants of food choice (i.e., attitude, self-efficacy, and intention) and if they used the logos to make healthy choices during lunch (in response categories ranging from one (= never) to five (= always) [23]. Finally, in two studies, measurements were conducted by qualified staff [25,26].

Outcomes included dietary intake (fruits and vegetables, fat and cholesterol), determinants of dietary behavior (self-efficacy, intention and attitude), body composition (body weight, body fat %, visceral fat), clinical biomarkers (blood sugar, LDL cholesterol), physical activity, mental health, and smoking.

Of the nine studies, three showed improvements in dietary practices [10,23,29], including increased fruit and vegetable consumption and lower fat, cholesterol, and energy intake.

A positive effect on dietary behavior was found in three studies [10,24,28]. In the first study, a significant effect was found on the perceived social support from colleagues regarding eating less fat [24]. In the second, it was shown that there were significant improvements in the perceived benefits of healthy nutrition practices and nutrition knowledge related to cardiovascular disease and cancer [10]. Finally, in the third study, in which employees were divided into three groups (two intervention and one control), the two intervention groups were more likely to report making lifestyle changes [28].

Five studies found a significant effect on health-related outcomes including weight loss, BMI reduction, abdominal circumference, lower blood sugar, blood pressure, metabolic syndrome markers, LDL and total-cholesterol [6,25,26,27,29].

#### 3.2.4. Multicomponent Interventions

Five studies provided a combination of components (i.e., multicomponent) including information, motivational prompts, and environmental changes [30,31,32,33,34]. Information was spread through the distribution of information sheets, brochures, leaflets, self-help manuals, posters, etc. [30,31,34], and educational programs [33], whereas environmental changes were carried out by increasing the availability of healthy foods [31,32]. Motivational prompts included footsteps on the floor and slim-making big mirrors [30], peer support in the form of modeling and education [32], workers’ participation in program planning [34], and comments and impressions from family members and the counselor about the progress of the participant [33]. Four studies included self-reports (dietary intake, physical activity) [31,32,33,34] and one [30] included data from measurements conducted by qualified staff (blood parameters).

Outcomes were overall dietary intake (e.g., fat, fruit, vegetables, high fat snack, alcoholic-drinks, sweet-drinks, grain and butter, margarine, dressing, mayonnaise, mushrooms, seaweed, etc.), body composition (subscapular skinfold thickness, waist circumference, body weight, BMI) and clinical biomarkers (diastolic and systolic blood pressure, total cholesterol, LDL, High-Density Lipoprotein (HDL), aspartate, insulin, plasma glucose, Homeostasis Model Assessment of Insulin Resistance changes (HOMA-IR)).

Improvements in diet were found in three studies [32,33,34]. Fruit consumption increased [32,34] as well as vegetable consumption [34] and high-fat snacks consumption decreased [32]. Moreover, consumption of white-vegetables, green/deep-yellow vegetables, mushrooms, seaweed, and konnyaku increased and consumption of alcoholic-drinks, sweet-drinks, large servings of grain and butter, margarine, dressing, and mayonnaise consumption decreased [33].

Positive health-related outcomes (decrease in LDL, total cholesterol, increase in the ratio between total and HDL–cholesterol (total/HDL), BMI, etc.) were reported in two studies [30,33].

## 4. Discussion

### 4.1. Overall Findings

The present literature review presents available studies implementing dietary workplace interventions among office workers. Four different intervention types were identified and these were web-based, food-based, information and multicomponent programs. The most commonly used designs were information and web-based interventions, which are the least expensive ways to intervene.

Food-based interventions increased the availability of healthy foods or provided ready-made meals. Although none of them aimed to change diet behaviors per se, all of them were successful at improving the participants’ dietary intake. However, one of them [22] aimed at reducing risk factors for the metabolic syndrome, with positive effects on blood pressure, serum lipids, and plasma ghrelin. Food-based interventions done in non-office settings report similar pattern of results [35,36]. For example, a study by Lachat et al. showed that providing fruits and vegetables in a university canteen contributed to a higher intake among students both at lunch and on an everyday basis [35]. Moreover, Backman et al. reported that blue collar workers who received fresh fruits and vegetables increased their consumption significantly compared to those in the control worksites [36]. The success of this type of intervention indicates that the provision of healthy food may be an easy and effective way to promote dietary changes.

Interventions that provided information only included nutrition counseling to help participants set priorities, establish goals, create individualized action plans and increase their awareness and knowledge about diet and health-related issues. This type of intervention was more effective at improving health-related outcomes than diet and dietary behavior. A potential explanation is that these interventions focus on improving both diet and physical activity, and part of the effect is from increased physical activity. A similar pattern of results was seen in an older study, not included in this review, that provided information about nutrition, which resulted in a reduction in weight and cholesterol among office workers [37]. Moreover, intervention studies done among non-office workers found that providing information resulted in positive changes. Briley et al. reported a reduction in weight and total cholesterol [38] and Aldana et al. demonstrated that educational courses in nutrition and physical activity had a beneficial effect and could potentially lessen the risks associated with common chronic diseases [39], thus confirming the results from the present review. Finally, most of the multicomponent interventions (a combination of healthy food, information, and motivational prompts) were more effective at improving dietary intake than health-related outcomes, and none of them aimed to change dietary behaviors. These interventions assessed a range of outcomes and the heterogeneity of reported findings made it challenging to summarize the results. Overall, positive effects in terms of increasing fruit and vegetable consumption, improved nutrient intake (e.g., decreasing fat and added sugars consumption, increasing fiber and protein consumption), increasing dietary knowledge, improving dietary behaviors (e.g., self-efficacy, planning healthy eating, attitudes towards healthy options) and aiding weight loss were reported. Improvements in health-related outcomes were also observed and were mainly in body composition and clinical biomarkers (total cholesterol, LDL, blood pressure, etc.).

Almost half of the multicomponent interventions focused on multiple health-related behaviors such as diet, physical activity, quitting smoking, reducing stress, etc., making it difficult to draw conclusions if dietary interventions alone or in combination with other health behaviors are more effective at improving health. Only a few studies aimed at improving diet exclusively, mainly by providing healthy food, with promising results. However, since these studies lasted between four weeks and 19,5 months, it is difficult to draw a conclusion regarding long-term effects.

### 4.2. Behavior Change Theories

Theories and models, relevant for the field of dietetics, are frameworks for helping practitioners understand external and internal issues and the dynamics that led to behavioral changes. The use of these frameworks provides a rationale for the selection of specific counseling strategies [40]. These theoretical frameworks are commonly used in intervention studies aimed at health promotion in workplaces. For example, the PACE (Physician-based Assessment and Counselling for Exercise) intervention aimed at enhancing moderate-intensity physical activity at the workplace was based on the TMBC and SCT [41]. Furthermore, in a review including twenty-one studies of worksite interventions addressing physical activity, behavior change theories were mainly used for counselling interventions and health education, with the TMBC being frequently applied [42]. Additionally, two large Dutch Randomized Controlled Trials (RCTs) which aimed at stimulating physical activity, embodied principles of cognitive behavioral therapy as well (e.g., modification of workers’ irrational beliefs about their back pain) [43,44]. However, in the present literature review, only half of the studies were designed around these theories [10,12,16,17,18,23,24,25,30,34].

### 4.3. Web Based

Most of the interventions that use behavior change theories and strategies to facilitate health and food behavior change were web-based. One of the most commonly used theories was SCT (also called social learning theory), which is based on the idea that people learn by observing other’s social interactions, experiences, and outside media influences (includes techniques such as modeling skill training, self-monitoring, and contracting) [45]. The results from interventions based on this theory are mixed, but encouraging results regarding improvements in dietary intake and dietary behavioral change [12,17] were found as were moderate effects for weight loss [16].

Another commonly used theory was the TMBC that describes a sequence of cognitive (attitudes and intentions) and behavioral steps people need to take to change behavior. The model offers effective and specific strategies at various points in the process of change and suggests outcome measures including decision balance and self-efficacy. Appropriate application of strategies is dependent upon the participant’s stage of change (precontemplation, contemplation, preparation, action, maintenance, and termination) [46]. Findings from the interventions based on this theory are promising with improvements in dietary intake and stages of readiness to eat healthier [17,18,30], and moderate with regards to weight loss [17].

### 4.4. Information

The theory of planned behavior (TPB) was one of the theoretical frameworks used among interventions that provided information as a mean to intervene. This theory suggests that the decision to behave in a certain way is the result of the likelihood of specific outcomes [40] and it was used successfully by Vyth et al. [23]. In their study, they added and labeled healthy options in worksite cafeterias expecting customers to increase healthy food consumption with the likelihood that they will benefit from this behavior.

Engbers et al. [24] applied the ‘attitude-social influence- (self-) efficacy model’ (ASE model) to measure psychosocial determinants of eating more fruit, and vegetables as well as less fat, and the model was also successful in affecting the reduction of fat intake. The ASE Model identifies the determinant ‘social influence’ instead of the determinant ‘subjective norm’. Social influence embraces subjective norms, social support/pressure, and modeling [47].

The Health Belief Model (HBM) was applied in a theory-based worksite intervention, conducted by Abood et al. [10] and it was successful at improving health beliefs, nutrition knowledge, and dietary behavior. The HBM implies that health behaviors are determined by health beliefs and readiness to act [48]. Constructs central to the HBM consist of perceived susceptibility, perceived severity, perceived benefits, perceived barriers, and other mediating variables. 

Improvements in metabolic risk factors were reported in one study conducted by Salinardi et al. [25] in which the SEM was used. The SEM is based on a theory related to the relationships between individuals, social groups, and the environment or community [40]. The SEM model was also applied in a large multicomponent randomized controlled trial conducted by Sorensen et al. [34] that included environmental aspects. The intervention comprised education, food tastings, family training, increased availability of fruit and vegetables, and food labeling. The study reported that the most intensive intervention (including the family component) was most successful and reported a significant increase in fruit and vegetable consumption.

### 4.5. Strengths and Limitations

A strength of the present literature review is the large number of articles included, of which most are randomized control trials and applied behavior change theories. However, there are limitations in this literature review that limit the generalizability of the results and the findings need to be considered with caution. Although we aimed to review dietary interventions per se in an office context, most of studies addressed a variety of health behaviors, making it difficult to disentangle the effect of dietary interventions alone. On one hand, improvements in diet could be linked to the dietary components such as environmental changes (e.g., provision of healthy food) and nutrition education and counseling. On the other hand, improvements could be linked to other parameters (physical activity, quitting smoking, reducing stress, etc.) that some of the studies focused on. Therefore, we cannot determine which of the above factors contributed exclusively to improvements in diet. The reviewed interventions were mainly carried out in the USA or Western Europe and these findings may not be applicable elsewhere. Bias regarding the target population may emerge. Only 10 of the 25 included studies specifically mention the term “office workers”, while, in the rest of them, office work was assumed by the reviewers, since the papers described the working place and not the people working there. The limitations of the individual interventions are also limiting the literature review in its conclusions, such as the self-reporting of dietary outcomes, short intervention duration, limited or no follow-up periods, lack of control group in some studies, small sample size and a heterogeneous distribution of gender (women are more interested in nutrition) and the health status (i.e., participants at high risk may be more likely to want to change). The lack of rigorous study design, i.e., non-randomized and non-controlled trials, was a limitation in several of the included studies. However, some authors argue that randomized controlled trials are not the most appropriate designs for dietary interventions and that researchers should aim to increase efficacy, reach and uptake of interventions [49]. Another limitation of the literature review involves the issue of effectiveness. This literature review attempted to assess which types of intervention were more effective without assessing the quality and the cost-effectiveness of the studies. In this literature review, effective study is defined as a study in which a study detected a change in at least one of the outcomes under consideration such as dietary intake.

### 4.6. Future Research

Due to the small number of studies focused exclusively on office workers, there is a need for further research to identify the effectiveness of dietary workplace interventions in this target group. Moreover, the specific characteristics of office workers, with mainly sedentary behavior at work, should be considered when creating tailored messages and interventions adapted to this type of work place. Moreover, methods to assess long-term health and economic benefits are needed, as well as methods to better understand the individual components for change in the intervention, i.e., the ‘how’, ‘why’ and ‘what’ [50]. Finally, future intervention studies should also be conducted over longer periods of time to assess the long-term changes regarding dietary intake and behavior.

## 5. Conclusions

Diet plays an important role in the prevention of NCDs, and workplace dietary interventions have the potential to improve many aspects of dietary habits, dietary behavior, and health outcomes. However, there is no ‘one design fits all’. Instead, one should focus on customized interventions, taking advantage of the specific work environment, such as interaction and support between colleagues, and taking into account eating habits inside and outside the office. Moreover, we suggest that future studies be more transparent when reporting results in terms of what did and did not work, as well as which approaches were well accepted by office workers. In this way, policymakers, employers and researchers can avoid making mistakes again.

## Figures and Tables

**Table 1:** Summary of the studies focusing on web-based interventions listed by author and year, the study design, duration, sample size, intervention type, intervention, incentive, outcome measures, and main findings.

Author (Year)	Location/Type of Work	Design/Duration	Population/Groups	Intervention/Theory	Incentive	Outcomes	Results
WEB-BASED (Educational Interventions)							
Cook et al. (2015) [12]	USA. Employees in offices of a technology company	Randomized Controlled Trial(RCT) 3months	Total *n*= 278 Intervention Group (IG) *n*= 138 Control Group (CG) *n*= 140	Web-based Health Promotion Program(HPP) for older adults (HealthyPast50) Provision of information/educational materials Social Cognitive Theory (SCT)	United States Dollar (USD) 50 to participate plus a USD 500 raffle drawing	1.Diet 2. Exercise 3. Stress 4. Aging beliefs 5. Tobacco use	IG vs. CG: Diet: IG showed significant improvement on dietary behavioral change self-efficacy (*p* = 0.048) and planning healthy eating (*p* = 0.03) Exercise: IG showed significant improvement in mild exercise (*p* = 0.01) Stress, Aging beliefs, Tobacco use: No difference Analysis when non-users were excluded *(used the program less than 30 min in total)*: IG showed significant improvement in: Diet: Eating practices (*p* = 0.03) Exercise: Exercise self-efficacy (*p* = 0.03), exercise planning (*p* = 0.03) Aging beliefs: improved (*p* = 0.01)
Sternfeld et al. (2009) [13]	USA. Employees in the administrative offices of a healthcare Organization	RCT 16 weeks	Total *n*= 787 IG *n*= 351 CG *n*= 436	ALIVE: An e-mail program, which offered: -Individually tailored small-step goals -A personal homepage with tips -Educational materials -Tracking and simulation tools 3 paths (1) Increasing Physical Activity (PA) (2) Increasing Fruits and Vegetables (F&V) (3) Decreasing fats and sugar	No monetary incentive, but those who completed the assessment received feedback regarding their current diet and physical activity	Changes in: 1. Diet 2. PA	The whole IG (3 paths) vs. CG: IG performed significantly better than CG Diet: Significant increase was observed in: - F&V consumption by 0.18 cup-equivalents/day (*p* = 0.03) Significant decreases were observed in: - Saturated fat consumption by 0.95 g/day (*p* = 0.01) - Trans-fat consumption by 0.29 g/day (*p* = 0.02) PA: Significant increases were observed in: - Moderate Physical Activity (MPA) by 28.0 min/week. (*p* = 0.0002) - Vigorous Physical Activity (VPA) by 12.5 min/week. (*p* = 0.03) - Walking by 21.5 min/week. (*p* = 0.0003) Significant decrease was observed in: - Sedentary behavior by 59.8 min/week (*p* = 0.05) The largest changes were in those who did not meet behavioral recommendations at baseline (increase of 55.4 min/week. of MPA and decrease of 1.15 g/day of trans fats) relative to the CG The improvements in diet and PA tended to maintain in the intervention group 4 months after the intervention ended
Perez et al. (2009) [14]	USA. State Health and Human services employees	Pre-post design 1 year	Total *n*= 1017 No control group	Web-based worksite wellness incentive program The Healthy Employee Lifestyle Program (HELP) Behavior change through 3 main approaches: (1) Providing an overall wellness report with tips for improving health (2) Rewarding health behaviors with points redeemable for incentives (3) Providing education and peer support. Transtheoritical Model of Behavior Change (TMBC)	Rewards for reporting health behaviors (e.g., t-shirts, water bottles, and up to 3 days of paid leave.)	1. F&V consumption 2. PA 3. Smoking 4. Age-appropriate health screenings 5. Weight management	Within IG: between baseline and follow-up (No control group) Diet: - More participants reported eating 3 or more F&V/day (*p* = 0.03) - Participants progressed in stages of readiness to change for eating 5 or more F&V/day (*p* = 0.002) and for eating a low-fat diet (*p* = 0.04)
Thomson et al. (2018) [15]	USA. Federal workplace researchers and support staff (laboratory, field and office)	Pretest-posttest 12 weeks	Total *n*= 22 No control group	“Nutrition 4 Weight Loss Program” Online nutrition education program for weight loss in the workplace Provision of information, educational materials, provision of eating plan	Access to the program was the only incentive for participants.	1. Anthropo-metric measurements body composition, blood pressure (BP), and skin carotenoid level (biomarker for F&V intake) 2. Feasibility components	Within IG: between baseline and follow-up (No control group) Anthropometric measurements: Significant decreases were observed in: -Diastolic Blood Pressure(DBP) (mean 3.6 mm Hg) (*p* = 0.01) -Weight (mean 1.8 kg) (*p* = 0.02) -Body Mass Index (BMI) (mean 0.6 kg/m^2^) (*p* = 0.01) -Body Fat Percentage (BFP) (mean 1.6%) (*p* = 0.003) -Visceral fat level (mean 0.7 cm^2^) (*p* = 0.02) Feasibility components: The program components reported to be the most liked were the class videos (64%) and the nutrition consultation (50%) whereas the least liked and used component was the food diary (14%)
Mouttapa et al. (2011) [16]	USA. Employees from two Southern California Universities	RCT 5 weeks and 2-month follow-up self-report assessments	Total *n*= 261 IG *n*= 118 CG *n*= 143	Personal Nutrition Planner (PNP), an online nutrition intervention tool, which calculate participants’ BMI, estimate energy expenditure and provide them with nutrition information IG: They registered on the site, completed the PNP and indicated if they wanted to receive weekly e-mail reminders (goals, steps and additional information). CG: They completed all the assessments like the IG. They were not provided with any health-related information, but they would receive access to the PNP after the study was completed. SCT		1.Dietary intake frequencies 2. Weight loss 3. Opinions regarding intervention	IG vs. CG: IG performed significantly better than CG Dietary intake: Increase in weekly dairy intake from nearly 9 times per week (pretest) to nearly 11 times (2 months posttest) (*p* < 0.05) Weight loss: Among participants who wanted to lose weight, weight loss in the IG was significantly higher than that of the CG. (*p* < 0.05) Opinions regarding the PNP intervention: On a scale of 1–5, mean ratings of the PNP program characteristics ranged from 3–4.
Cook et al. (2007) [17]	USA. Employees in offices of a Human resources company	RCT 3 months	Total *n* = 419 IG *n*= 209 CG *n* = 210	Comparison of a multimedia Web-based program with printed materials IG: Web-based program with information and guidance on the major health promotion and wellness topics of stress, nutrition/weight management, and fitness/PA CG: Printed materials covering the same health topics as the Web-based program (but not necessarily the same content) SCT, TMBC	USD 50/survey and a USD 500 raffle prize	1. Dietary measures 2. Stress Management 3. PA 4. Body Weight (BW)	IG (Web-based group) vs. CG: (printed materials) Dietary measures: Significant positive effects on attitudes Toward a Healthful Diet and Dietary Stage of Change in the IG BW: No significant differential change in weight between the two groups. Although both groups reported weight loss. (IG: *p* = 0.04, CG: *p* = 0.02) Stress Management: No differences between the two groups PA: No differences between the two groups Within IG (Web-based group) Significant positive effects regarding the number of times the subject accessed the program about measures of: -Dietary Self-Efficacy (*p* = 0.003) -Attitudes Toward a Healthful Diet (*p* = 0.045) -Dietary Stage of Change (*p* = 0.005)
Papadaki et al. (2005) [18]	Scotland. Universities of Glasgow and Glasgow Caledonian/ Female university workers	Quasi-experimental 6 months	Total *n*= 72 Dietary analyses: IG *n*= 53 CG *n*= 19 Analysis of biomarkers: IG *n*= 46 CG *n*= 16	Provision of internet education via an innovative Mediterranean Eating Website IG: Tailored dietary and psychosocial feedback and Internet nutrition education CG: Minimal dietary feedback and general healthy-eating brochures Precaution Adoption Process Model (PAPM), TMBC		1.Diet 2. Clinical and anthropometric measurements	IG vs. CG: Diet: Intake of nuts, fruits and seeds was significantly - increased by 34,9 g/day (*p* = 0.022) in the IG - decreased by 23.2 g/day (*p* = 0.022) in the CG Intake of dairy products was significantly - decreased by 4.1 g/day (*p* = 0.033) in the IG - increased by 42.3 g/day (*p* = 0.033) in the CG Intake of cereals - decreased by 15.6 g/day (*p* = 0.059) in the IG - increased by 14.3 g/day (*p* = 0.059) in the CG Clinical and anthropometric measurements: No significant differences. However, IG showed a significantly higher increase in HDL-cholesterol compared with CG (0.22 mmol/l vs. 0.06 mmol/l) (*p* = 0.036) as well as a higher decrease in the ratio of total: High-Density Lipoprotein-cholesterol (HDL) (−0.44 vs. −0.04) (*p* = 0.008) Within IG: between baseline and follow-up Increases in intake of: - Vegetables by 0.5 servings/day - Fruits by 0.4 servings/day - Legumes increased from 15.9 g/day to 30.6 g/day -Monounsaturated Fatty Acids (MUFA): saturated fatty acid (SFA) ratio increased from mean 1.49 to 1.79
Bennett et al. (2011) [19]	USA. Managers from eight organizations	RCT 6 months	Total *n* = 109 IG *n* = 47 CG *n* =62	The Internet-based program *ExecuPrev™*, trained managers to modify attitudes and behaviors, and built motivation to be healthy and effective leaders. IG: Health education (on diet, exercise, and stress) and leadership development exercises to enhance perceived career benefit of participating (lessons, webinars, additional links and interactive multi-media lessons). CG: No program		1.Diet 2. Exercise 3. Mental health 4. Biometric measurements	IG vs. CG: IG performed significantly better than CG Diet: Significant improvements in dietary attitudes and dietary self-efficacy (*p* = 0.00) Exercise: Marginally significant improvements in frequency (*p* = 0.07) Mental health: Significantly fewer distress symptoms (*p* = 0.01) Biometric Changes: Women: Significant reduction in WC for the IG compared with CG (*p* = 0.02). IG lost about 1.26 inches more from their waists than CG.

**Table 2:** Summary of the studies focusing on the provision of healthy food listed by author and year, the study design, duration, sample size, intervention type, intervention, incentive, outcome measures, and main findings.

Author (Year)	Location/Type of Work	Design/Duration	Population/Groups	Intervention/Theory	Incentive	Outcomes	Results
Provision of Healthy Food (Environmental Interventions)							
Lassen et al. (2012) [20]	Denmark. Financial worksite	Intervention without a control group (CG) 7 weeks	Total *n*= 27 Participants served as their own control by comparing nutritional intake on days receiving Canteen Take Away (CTA) meals with days not receiving CTA	Provision of a free CTA, a healthy ready-to-heat meal (10 different meals) on two weekdays for employees as well as for their families	Employees were paid for time off work to receive instruction and to complete the dietary interviews.	The effectiveness of a CTA concept in promoting healthy eating habits among employees	CTA days vs. non-CTA days: (No control group) CTA consumption showed nutritional benefits Intake of F&V was on average 129 g higher on CTA days (*p* = 0.002) Most of the difference in Fruits and Vegetables (F&V) intake was accounted for by an increase of vegetable intake by 109 g, equaling about 1 serving. Average energy density on CTA days (excluding beverages) was 77 kJ/100 g lower than the non-CTA days. (*p* = 0.01) Energy percentage of protein was on average 2.7% higher (*p* < 0.001)
Alinia et al. (2011) [21]	Denmark. Workplaces with mainly white-collar workers	Non-Randomized Controlled Trial (RCT) 5 months	Total *n*= 124 Intervention Group (IG) *n*= 68 CG *n*= 56 Total sites = 8 Intervention (I) sites *n*= 5 Control (C) sites *n*= 3	IG: Fruit basket with free fruits (mainly apples, pears, oranges and bananas) CG: No fruit basket		1. Fruit consumption	IG vs. CG: Fruit intake: Significantly increased in IG (*p* = 0.021) Within IG: Between baseline and follow-up Dietary intake: mean daily fruit consumption increased significantly by 112 g (*p* = 0.002) mean daily dietary fiber consumption increased significantly by 3 g (*p* = 0.007) mean daily added sugar consumption significant decreased by 10,7g (*p* = 0.019)
Inoue et al. (2014) [22]	Japan/ Office workers in the city hall with mostly low levels of Physical Activity (PA)	Non-RCT 3 months	Total *n*= 35 IG *n*= 28 CG *n*= 7 Participants were able to self-select the control or intervention group.	Provision of a Japanese-style healthy lunch at the workplace cafeteria to provide balanced nutrition and sufficient vegetable consumption IG: Japanese-style healthy lunch. 2 subgroups (1) Intake frequency less than 50 meals out of the total 61 meals (<50/61) (2) Intake frequency more than 50 meals out of the total 61 meals (>50/61) CG: Consumed their habitual lunches without restriction		1. Dietary intake 2.Blood parameters 3.Anthropometric data	Within IG: between baseline and follow-up Dietary intake: IG (< 50 out of 61 meals): Energy and carbohydrate intake significantly decreased (energy: 2554 ± 392 kcal vs. 2104 ± 393 kcal, *p* = 0.042; carbohydrate: 359.6 ± 85.2 g vs. 295.8 ± 45.3 g). IG (> 50 out of 61 meals): Total dietary fiber and total vegetables significantly increased (total dietary fiber: 15.3 ± 5.2 g vs. 30.4 ± 20.9 g, *p* = 0.047; total vegetables: 292.4 ± 146.6 g vs.411.1 ± 155.9 g, *p* = 0.035). Blood parameters: IG (<50 out of 61 meals):Plasma active ghrelin and desacyl ghrelin levels significantly increased (active ghrelin: 1.4 ± 2.0 fmol/mL vs. 3.8 ± 3.9 fmol/mL, *p* = 0.008; desacyl ghrelin: 41.6 ± 49.0 fmol/mL vs. 101.4 ± 89.3 fmol/mL IG (>50/6150 out of 61 meals):T-Chol, Low Density Lipoprotein (LDL), levels significantly decreased (T-Chol: 211 ± 27 mg/dL vs. 199 ± 22 mg/dL, *p* = 0.006; LDL: 127 ± 31 mg/dL vs. 116 ± 25 mg/dL, *p* = 0.010) and plasma active ghrelin and desacyl ghrelin levels significantly increased (active ghrelin: 1.9 ± 5.9 fmol/mL vs. 5.3 ± 8.4 fmol/mL, *p* = 0.001; desacyl ghrelin: 77.4 ± 135.4 fmol/mL vs. 115.7 ± 180.7 fmol/mL). Anthropometric data: IG (<50 out of 61 meals): Diastolic Blood Pressure (DBP) significantly decreased (90.5 ± 11.9 vs. 86.3 ± 11.4, *p* = 0.000). IG (>50 out of 61 meals): Body Fat Percentage (BFP), Systolic Blood Pressure (SBP), DBP significantly decreased (BFP: 23.8 ± 3.5 vs. 22.7 ± 3.6, *p* = 0.019; SBP: 137.5 ± 15.0 vs. 131.9 ± 16.9, *p* = 0.023; DBP: 88.4 ± 10.6 vs. 80.8 ± 8.7, *p* = 0.000). The results grew more pronounced as intake of Japanese-style healthy lunches increased in frequency Within CG: between baseline and follow-up Dietary intake: The “other vegetable” intake significantly decreased (240.1 g ± 128.5 g vs. 96.4 g ± 64.7 g, *p* = 0.015). Blood parameters: Hemoglobin A1c (HbA1C) levels had significantly increased (4.99% ± 0.29% versus 5.13% ± 0.21%, *p* < 0.05) Anthropometric data: No significant differences

**Table 3:** Summary of the studies focusing on the provision of information listed by author and year, the study design, duration, sample size, intervention type, intervention, incentive, outcome measures, and main findings.

Author (Year)	Location/Type of Work	Design/Duration	Population/Groups	Intervention/Theory	Incentive	Outcomes	Results
Provision of Information (Educational or Environmental Interventions)							
Vyth et al. (2011) [23]	Netherlands. Office workers with mainly sedentary jobs, worksite cafeterias	Randomized Controlled Trial (RCT) 3-week was repeated 3 times during the 9-week research period	Total *n*= 368 Intervention Group (IG) *n*= 232 Control Group (CG) *n*= 136 Total cafeterias = 25 Intervention (I) cafeterias *n* = 13 Control (C) cafeterias *n* = 12	IG: Healthy options added and labelled CG: Same menu without the logo Theory of Planned Behavior (TPB)		1. Sales data: sandwiches, soups, fried snack foods, fruit, salads 2. Behavior determinants	IG vs. CG: Sales data: Fruit sales were significantly higher in the IG (*p* = 0.001). This effect represents 1 c fruit per 50 employees per week. This change continued during the post-intervention period Behavior determinants: No significant differences
Engbers et al. (2006) [24]	Netherlands. Two companies with office workers	Controlled Longitudinal Trial (CLT) 12 months	Total *n*= 432 IG *n* = 191 CG *n* = 241 Total sites = 2 I sites *n*= 1 C sites *n*= 1	The FoodSteps intervention Food part: Placement of informational sheets near food products, to stimulate healthier food choices at company’s canteen Physical Activity (PA) part: (i.e., stimulating stair-use) ASE model: Attitude-social influence- (self-)efficacy model’		1.Psychosocial determinants of behavior in: 1. Fruit and Vegetables (F&V) consumption 2. Fat consumption	IG vs. CG: Psychosocial determinants of behavior in: F&V consumption: No effects were found Fat consumption: At 3 months a significant positive effect was found on the perceived social support from colleagues regarding eating less fat in the IG, but at 12 months the attitude and self-efficacy towards eating less fat became less positive. Self-efficacy towards eating less fat at work decreased significantly in the IG. This effect was also found at 12 months.
Abood et al. (2003) [10]	USA. Administrative staff at a university	Pretest-posttest 8 weeks	Total *n*= 53 IG *n*= 28 CG *n*= 25	*Theory-based, worksite-tailored nutrition education program* IG: Eight 1-h weekly educational sessions. CG: Subjects did not receive any form of intervention. They received an abbreviated version of the nutrition education intervention 1-month post intervention. Health Belief Model (HBM)		1. Health beliefs and nutrition knowledge 2. Dietary behaviors	IG vs. CG: Health beliefs and nutrition knowledge: Significantly improvements in perceived benefits of healthy nutrition practices and nutrition knowledge related to cardiovascular disease and cancer in the IG. (*p* < 0.001) There was also an association between nutrition knowledge and higher fiber intake (*p* < 0.005) and between nutrition knowledge and consuming a lower energy percentage of total fat and saturated fat (*p* < 0.005) Dietary behavior: Participants significantly reduced (each *p* < 0.001) Total calories: by approximately 840 kcal/day Fat intake: by 45 g/day Saturated fat: by 18 mg/day Cholesterol intake: by 158 mg/day,
Salinardi et al. (2013) [25]	USA. Worksites office-based companies	RCT 12 months	Total *n* = 133 IG *n*= 94 CG *n* =39 Total sites = 4 I sites *n*= 2 C sites *n*= 2	Weight loss and maintenance program (6 months each) IG: nineteen 1-h long education sessions, health and nutrition education program open to all employees (newsletters on healthy eating and seminars) and a 6-months structured maintenance program was also offered to employees who completed the weight-loss program CG: no intervention Social Ecological Model (SEM)		1.Body weight 2. Cardio-metabolic risk factors	IG vs. CG: There were significant improvements in the IG Cardiometabolic risk factors: Fasting total cholesterol, glucose, Systolic Blood Pressure (SBP) and Diastolic Blood Pressure (DBP) were significantly improved (*p* ≤ 0.02 for each) Body weight: The weight loss in kg in IG (−8.0 ± 0.7;) was significantly different from the CG (weight gain +0.9 ± 0.5 kg) *p* < 0.001) after 12 months.
Allen et al. (2012) [26]	USA. Employees of the University of New Hampshire	Semi-RCT 12 months	Total *n*= 55 IG *n*= 26 CG *n*= 29	Comparison of health risk factors of employees who received health risk screening plus lifestyle education (IG) with those who received screening plus minimal information (CG). IG: monthly education sessions and pedometers CG: no intervention		1.Anthropometric Body Mass Index (BMI), Waist Circumference (WC), Body Fat Percentage (BFP) 2.Clinical measures: Low-Density Lipoprotein, (LDL), total cholesterol (T-Chol)	IG vs. CG: Clinical measures: At 12 months, LDL (*p* = 0.01), T-Chol (*p* = 0.01) and several metabolic syndrome markers (*p* = 0.002) were significantly lower in the IG than in the CG (LDL: 110.9 mg/dL vs. 126.7 mg/dL and T-Chol: 183.4 mg/dL vs. 198.6 mg/dL) Within groups: between baseline and follow-up Anthropometric: CG: Waist Circumference (WC) increased (37.1 to 38.9 inch), (*p* < 0.05)
Nisbeth et al. (2000) [27]	Denmark. White collar workers employees in a computer company	RCT 1 year	Total *n*= 74 IG *n* = 48 CG *n* = 26	Counselling on diet, exercise and smoking and its effect on Coronary heart disease (CHD) risk factors IG: divided into3 subgroups: 1. Exercise group (EG) aerobic exercise 3 times/week 2. Diet group (DG) reduce intake of saturated fat and increase fish products 3. Smokers group (SG) quit smoking The subjects could participate in more than one intervention CG: No intervention		1.Lifestyle changes 2. CHD risk factors	All 3 IG groups vs. CG: Significant differences in favor of the IG: Lifestyle changes: The fitness level (aerobic power) increased (*p* < 0.01) CHD risk factors: BW and BMI decreased (*p* < 0.05) DG vs. CG: Compared to the CG beneficial changes were found in BW, total cholesterol, LDL, Triglycerides and LDL/High-Density Lipoprotein (HDL) ratio. SG vs. CG: Compared to the control group changes were found in total cholesterol, LDL and LDL/HDL ratio. Within IG: between baseline and follow-up EG: Aerobic power increased from 37.0 to 40.5 mL min^−1^ kg^−1^ (*p* < 0.01) DG: HDL increased from 1.12 to 1.27 mmol L^−1^ (*p* < 0.01), Tr decreased from 2.10 to 1.50 mmol l^-1^ (*p* < 0.05) and LDL/HDL ratio decreased from 3.86 to 3.32 (*p* < 0.05). SG: HDL increased from 1.10 to 1.23 mmol L^−1^ (*p* < 0.01), LDL/HDL ratio decreased from 3.43 to 2.97 (*p* < 0.05), DBP increased from 75 to 78 mmHg (*p* < 0.05) and aerobic power increased from 2.96 to 3.07 L min^−1^ (*p* < 0.05).
Addley et al. (2014) [28]	UK. Department of Finance and Personnel (DFP) within the Northern Ireland Civil Service (NICS)	RCT 12 months	Total *n*= 132 IG *n* = 84 CG *n* = 48	IG: divided into 2 subgroups: Groups A: Health risk appraisals (HRA) augmented with health promotion and education activities Group B: HRA only CG: Group C		1. Lifestyle parameters (BMI, alcohol, PA) 2. Mental health 3. Work ability 4. Self-perceived health behavior change (healthy diet)	IG vs. CG: Lifestyle parameters (BMI, alcohol, PA), Mental health and Work ability: No effect Self-perceived health behavior change (healthy diet): Groups A and B were more likely to report making a lifestyle change in comparison with the CG. Within IG (Group A vs. Group B) Group A was considerably more likely to report a change compared with Group B
Horan et al. (2018) [29]	USA. University Employees	Pretest-Posttest 10 weeks	Total *n*= 24 No control group	10-week session groups - Weekly meetings of didactic psycho education (30 min) and group exercise (30 min) - Weekly workbook activities (goal setting, nutrition, physical activity etc.) - Optional individual health coaching sessions	Participants submitted a United States Dollar (USD) 100 deposit, which was returned based on participation and completion of program requirements	1. Dietary intake 2. Anthropometric data 3. Physical fitness 4. Mindfulness and self-compassion	Within IG: between baseline and follow-up (No control group) Dietary intake: Fat consumption significantly decreased (*p* = 0.019) and mindful eating increased (*p* = 0.001) Anthropometric data: Abdominal circumference significantly decreased (*p* = 0.01), and thigh circumference significantly increased (*p* < 0.001). Physical fitness: Muscular endurance (crunches and push-ups) (*p* = 0.015, *p* = 0.008), leisure time PA (*p* = 0.034) and mindful exercise increased (*p* < 0.001), and physical well-being improved (*p* = 0.005). Mindfulness and self-compassion: Self-compassion (*p* < 0.001) and well-being (*p* = 0.003) improved
Kim et al. (2012) [6]	Korea. Office workers with abnormal findings	Before-after 4 months	Total *n*= 75 male workers	Workplace-visiting nutrition education program Anthropometric and clinical measurements Consulting of examination results Nutrition education Assessment		1.Anthropometric data 2.Clinical measures	Within IG: between baseline and follow-up (No control group) Anthropometric data: BMI was significantly reduced from 25.7 kg/m^2^ to 25.4 kg/m^2^ (*p* < 0.05) Clinical measures: Significant decrease in: - Fasting blood sugar: from 100.5 mg/dL to 97.0 mg/dL (*p* < 0.01) - T-Chol: from 211.3 mg/dL to 204.4 mg/dL (*p* < 0.05) - LDL: from 131.1 mg/dL to 123.6 mg/dL (*p* < 0.05)

**Table 4:** Summary of the studies focusing on multicomponent interventions listed by author and year, the study design, duration, sample size, intervention type, intervention, incentive, outcome measures, and main findings.

Author (Year)	Location/Type of Work	Design/Duration	Population/Groups	Intervention/Theory	Incentive	Outcomes	Results
Multicomponent							
Engbers et al. (2007) [30]	Netherlands. Two companies with office workers	Controlled Cluster Trial (CCT) 12 months	Total *n*= 452 Intervention Group (IG) *n* = 205 Control Group (CG) *n* = 247 Total sites = 2 Intervention (I) sites *n*= 1 Control (C) sites *n*= 1	‘Food’-part: to stimulate healthier food choices by means of product information in the canteen ‘Steps’-part: focused on stimulating stair use by means of motivational prompts in staircases and on elevator doors.		1. Biological cardiovascular risk indicators	IG vs. CG: Significant differences in favor of the IG: - Total cholesterol for women decreased by 0.35 mmol/L (*p* = 0.001) -High-Density Lipoprotein (HDL) for men increased by 0.10 mmol/L(*p* < 0.001) - The total cholesterol (T-Chol)–HDL ratio for the total group decreased by 0.45 mmol/L (*p* < 0.001) A difference in change in Systolic Blood Pressure (SBP) was found in favor of the CG (~4 mm Hg), due to an increase in the IG. Both groups: showed a decrease in all body composition values
Steenhuis et al. (2004) [31]	Netherlands. Companies and governmental organizations with mainly white-collar workers, worksite cafeterias	Randomized Controlled Trial (RCT) (companies were randomized) 6 months	Total *n*= 1013 IG *n* = 798 CG *n* = 215 Total work site cafeterias *n*= 17	4 conditions: (1). Educational Program (EP) (information about healthy nutrition) (2). Food supply Program (FSP) (increased availability of low fat products and fruits and vegetables) + EP (3). Labeling Program (LP) (low-fat products were labeled) + EP (4). No Program (NP)	People could obtain the self-help manual for free by filling out a coupon that was included in the brochures.	1. Sales data: low-fat milk, butter, meat, cheese products and desert 2. Food intake	LP vs. EP: Sales data: In the LP sales of low-fat desserts were increased (*p <* 0.01) LP vs. NP: Sales data: In the LP sales of low-fat desserts were increased (*p* < 0.05) Food intake: Total fat intake was decreased for respondents who believed they ate a high-fat diet (*p* = 0.04).
Hutchinson et al. (2013) [32]	Australia. Utility company	Non-randomized Observational Study (NROS) 4 weeks	Total *n*= 75 IG *n*= 54 CG *n*= 21 Total work sites *n*= 3	Group A: Provision of free fruit Group B: Free fruit and peer education/modelling condition Group C: Control group	Peer educators (Employees) received a small financial reimbursement for their time	1. Dietary intake (consumption of fruits and high fat snacks at work and home)	Dietary intake: *Fruits:* Fruit consumption significantly increased at work (*p* = 0.04) A vs. B: fruit consumption in Group B was greater than that in Group A B vs. C: fruit consumption in Group B was greater than that in Group C It was more successful among those who were not meeting the recommendations (2 weeks post-intervention the change was not maintained *p* = 0.76) High fat snacks A vs. C: Group A decreased the number of high fat snacks consumed pre- to post-intervention and Group C increased high fat snack consumption B vs. C: Group B decreased the number of high fat snacks consumed pre- to post-intervention and Group C increased high fat snack consumption. Fat consumption was decreased to a greater extent in those who were not already eating two serves of fruit per day. Only Group B managed to maintain this change *(p = 0.05).*
Maruyama et al. (2010) [33]	Japan. Office workers of the Nichirei Group Corporation with MetS risk factors	RCT 4 months	Total *n*= 101 IG *n* = 52 CG *n* = 49	LiSM10! program composed of individual structured counseling sessions, social and environmental approaches. Individual counseling: Monthly individual contact with a dietitian and a physical trainer. Goal setting: After baseline assessment, the participants attended an individual goal and action planning session and they reviewed their plans with counselors		1. Diet 2. Physical Activity (PA) 3. Metabolic parameters	IG vs. CG: Significant differences in favor of the IG: Diet: Intake of habitual food group significantly changed (recommended food consumption increased and consumption of foods to avoid decreased) (*p* = 0.00). PA: No difference between the two groups in the number of steps Metabolic parameters: Body Weight (BW), Body Mass Index (BMI), fasting plasma glucose, insulin, aspartate aminotransferase (*p* < 0.05), homeostasis model assessment of insulin resistance changes (HOMA-IR) significantly improved (*p* < 0.01)
Sorensen et al. (1999) [34]	USA. White collar workers in community health centers	RCT 19.5 months	Total *n*= 1306 (22 worksites)	3 intervention groups: (1). Minimal intervention (CG) (2). A worksite intervention (3). A worksite plus family intervention Social Ecological Model (SEM)		1. Diet (Fruit and Vegetables (F&V), fat, fiber consumption)	Within all groups: between baseline and follow-up Diet: F&V intake increased by: 7% in the worksite intervention group (approx. 0,2 servings) 19% in the worksite-plus-family group (approx. 0,5 servings) 0% in the control group (1 vs. 2 vs. 3) 1 vs. 2: The difference between the worksite intervention and the CG is not statistically significant (*p* = 0.47) 1 vs. 3: The increase in the worksite plus family group is significantly greater than that in the CG (*p* = 0.02). These changes reflect an increase of half a serving compared to the CG (*p* = 0.018) 2 vs. 3: The overall difference in F&V intake among the intervention groups is statistically significant (*p* = 0.05)

Abbrevations: ALIVE = A Lifestyle Intervention via E-mail, BFP = Body Fat Percentage, BMI = Body Mass Index, BP = Blood Pressure, BW = Body Weight, C sites = Control sites, CCT = Controlled Cluster Trial, CG = Control Group, CHD = Coronary Heart Disease, CLT = Controlled Longitudinal Trial, DBP = Diastolic Blood Pressure, EP = Educational Program, F&V = Fruit and Vegetables, fmol/mL = femtomole/milliliter, FSP = Food Supply Program, g/d = gram/day, g = gram HbA1c = Hemoglobin A1c, HBM = Health Belief Model, HDL = High-Density Lipoprotein, HPP = Health Promotion Program, I sites = Intervention sites, IG = Intervention Group, kcal = kilocalories, kg = kilogram, L = litre, LDL = Low-Density Lipoprotein, LiSM10! = Life Style Modification Program (individually tailored behavior change-oriented program, in the workplace), LP = Labeling Program, MetS = Metabolic Syndrome, mg/d = milligram/day, mg/dL = milligram/deciliter, min = minutes, mL = milliliter, mmHg = millimeters of Mercury, mmol = millimole, MPA = Moderate Physical Activity, MUFA = Monounsaturated Fatty Acids, NP = No Program, Non-RCT = Non-randomized Controlled Trial, NROS = Non-randomized Observational Study, PA = Physical Activity, PAPM = The Precaution Adoption Process Model, RCT = Randomized Controlled Trial, SBP = Systolic Blood Pressure, SCT = Social Cognitive Theory, SEM = Social Ecological Model, SFA = Saturated Fatty Acids, T-Chol = Total cholesterol, TMBC = Transtheoretical Model of Behavior Change, TPB = Theory of Planned Behavior, Tr = Triglyceride, USD = United States Dollar, VPA = Vigorous Physical Activity, vs. = Versus, WC = Waist Circumference.
